# The Vomiting of Pregnancy and Its Treatment

**Published:** 1878-06

**Authors:** 


The Vomiting of Pregnancy and its Treatment.—Although a secondary or reflex manifestation, the vomiting of pregnancy is of such frequent occurrence, and often obstinate persistence, as to have acquired a name and a place in medical literature.

We know that pregnancy, in perhaps a large majority of cases, if, indeed, there is an exception, gives rise to morbid conditions of some organ or organs, continuing during a part, and sometimes the whole term, of gestation. There is a susceptibility of the system to excitement during pregnancy that does not exist at other periods, owing, no doubt, to the intimate connexion of the organs of generation with the cerebro-spinal and the ganglionic system of nerves. The functions of the brain, those of respiration, circulation, secretion, digestion, and nutrition, may one or all be disturbed by conception and development of a new life within the old.

The stomach is usually the first organ to sympathize, and it is generally independent of any noticeable change of temperature or disturbance of circulation. This sympathy of the stomach is of various degrees of intensity, from a fastidious taste and appetite to nausea and vomiting. The period after, and the length of time it continues, vary in different individuals as well as in the same subject in succeeding pregnancies. While in some persons the nausea, or morning sickness, as it is sometimes called, commences almost immediately after conception, with the majority it does not begin until from the third to the fourth or sixth week of gestation, and usually terminates at about the fourth month. It sometimes continues more or less severe until the termination of gestation. There are others in whom this reflex disturbance is not severe until the fifth, sixth, or seventh month of utero-gestation, and yet others who are free from this sickness throughout the whole period of pregnancy.

The violence and frequency of the vomiting are sometimes so intense and persistent as to destroy the life of the patient. Cases have been reported where, from the inability of the stomach to retain the least particle of nourishment, death has resulted from starvation. Dr. Marshall Hall speaks of a case which occurred under his notice, but not in his care, in which the vomiting continued in spite of every remedy which intelligence could suggest, and which terminated fatally at the seventh month. The reported cases are numerous where death was averted either by spontaneous or induced labor.

A case is reported in *The Lancet* for 1838, of a lady who soon after her marriage ceased to menstruate, and became affected with morning sickness, which soon became so violent that nothing could be retained by the stomach. In this case, the report says, "the disorder was strangely attributed to disease of the pylorus. The sickness and extreme emaciation were the only symptoms present; after death no morbid appearances were found in any part of the body; a fœtus about four months old was in the uterus." This patient, it would seem from the foregoing statement, was literally starved to death. Dr. Davis, in his "Obstetric Medicine," relates similar cases. Dr. Dance, of Paris, reports a case that "during the second month after the arrest of the catamenia, was harrassed with almost constant vomiting, rejecting everything she took, whether liquid or solid, rapid emaciation following. Tongue clean and moist, no febrile symptoms present, no tenderness of the epigastrium on pressure, sleep interrupted, habitual constipation, vomiting both night and day. The matter ejected was of a greenish or limpid character, and small in quantity. The patient did not think herself pregnant, and there was no enlargement of the hypogastric region. All remedial measures were used without benefit; ice internally and externally, leeches, blisters, anti-emetic draughts, opium internally and externally, and twenty other remedies, without having the slightest effect in checking the vomiting. Emaciation in this patient by the end of May had made great progress; and now the hypogastrium began to be prominent, and pregnancy was not until then ascertained to exist, which was fully four and a half months. On the 2d of June she died."

I have quoted thus fully from this report, because it furnishes a very good type of the inveterate cases of vomiting during pregnancy. In this case the patient suffered almost from the beginning, vomiting continuing with increasing severity until death; almost five months pregnant. The report upon the *post mortem* examination says: "No lesion could be detected in the stomach; except a slight reddish tint of the mucous lining, the whole intestinal tube was sound. The uterus rose a few inches above the pubes, and its parietes were preternaturally soft and flabby, but without any other appreciable change of structure. The membranes of the fœtus were transparent throughout, but between these and the uterus were false membranes forming a layer some lines in thickness, exactly resembling those found between the pleura after inflammation; the same was found between the placenta and uterus, but more of a purulent character."

Another case, reported by M. Dance, did not reveal any products of inflammation between the uterus and membranes. Was not the uterine inflammation in the first case rather in consequence of, than the cause of, the violent and protracted vomiting? Would not inflammation producing such grave symptoms end in abortion or death before the expiration of four and a half months?

Pathologists (many, at least) attribute this reflex manifestation to the distension and development of the dense uterine structure after conception.

Dr. Graily Hewitt, however, in a paper read before the London Obstetrical Society in 1872, attributes the sickness and vomiting in pregnancy, in a majority of cases, to the irritation caused by flexion of the uterus, either ante or retro-flexion. Owing to the flexion the uterine fibres and nerves at that point are compressed, and this compression is increased up to a certain period, by the constantly increasing development of the gravid uterus; and when pregnancy advances to the fourth month or more, the flexion is more or less corrected by the natural rising of the uterus from the pelvic cavity, after which, he says, the sickness and vomiting generally subside. He believes this to be the "almost universal cause of vomiting in pregnancy." That the tissues of the uterus resist expansion is, he says, unquestionably the case, "but this is not enough, apart from the conjoined flexion of the organ, to account for more than a small number of cases. Dr. Hewitt says he has not had an opportunity of examining cases of vomiting in pregnancy after the fourth month, and is not sure how often vomiting is noticed in this degree after that period, and therefore cannot pronounce any opinion derived from actual observation as to the state of the uterus under such circumstances. He, however, admits that "there are probably a small number of cases in which the vomiting persists even after the flexion has been remedied by the gradual development of the gravid uterus." So far, he says, "as the pathology of this affection is concerned, the ordinary cases, where the vomiting is very slight, and hardly calls for medical attention, is due (in his opinion) to a temporary evanescent flexion of the uterus." M. Brian attributes the reflex irritation to anteversion or retroversion of the uterus. It is probable that these abnormal positions of the gravid uterus may aggravate the sickness, which is almost a concomitant of pregnancy, but are not the cause of it. Conception, perhaps, rarely occurs without being followed sooner or later by a sympathetic manifestation of some kind in some organ; no doubt, in many persons in so slight a degree as to escape special notice, but in a large majority sympathetic or reflex phenomena of various kinds, such as an undefinable sinking sensation about the epigastrium, a slight fullness of the head, dizziness, palpitation of the heart, oppression in breathing, loathing of food, heartburn, eructation (sometimes acid, sometimes not), nausea, vomiting, constipation, diuresis, headache, toothache, etc. And there are some women who know to a surety, by being troubled with some one or more of these manifestations, that they are pregnant. Disturbance of the stomach is, however, the most frequent reflex affliction of pregnancy. Several members of the London Obstetrical Society took exception to Dr. Hewitt's paper as to the cause of vomiting stated therein, saying they had known flexion and pregnancy coexisting without sickness, and, on the other hand, had frequently met with nausea and vomiting without flexion.

As to the treatment, we know how unsatisfactory have been our efforts to relieve this affection by the exhibition of drugs. I do not allude to the mild cases which require but little or no attention, but the excessive and persistent cases of vomiting, where the patient, in spite of all remedies, continues to grow, day by day, more feeble and emaciated. It is wonderful, the number of remedies which have been suggested by different authors and contributors. Purgatives, emetics, anti-emetics, vegetable and mineral acids, alkalies and antacids of various kinds, anti-spasmodics, narcotics of every variety, internally, externally, and hypodermically administered; tincture of iodine in minute doses, oxalate of cerium, effervescent nitrate of cerium, aconite, various effervescing draughts, bismuth, strychnia, etc., to end with varying success, sometimes with no success at all. Believing that the vomiting of pregnancy is a reflex phenomenon, is it not strange that nearly all our efforts to relieve it have been directed to the stomach, the helpless sufferer from the fault of another organ? Why not direct our curative measures directly to the source of mischief? Impressed with the correctness of this idea, I decided to put it in practice in the first case that might come under my care.

It has been now six years since my first opportunity of testing this idea, and within that time I have treated five cases, and in each case a very gratifying result ensued. I thought by exciting an irritation or superficial inflammation of the os and cervix uteri, the reflex nervous phenomena would be concentrated at the point of irritation, and thereby relieve the stomach.

To the first patient I applied the solid nitrate of silver to the os uteri only. The benefit was very noticeable within twenty-four hours. Being somewhat apprehensive, I applied the caustic rather sparingly, and in a few days applied it again, obtaining still greater relief. I used it a third time, but suspect the third application was really unnecessary. The patient remained free from sickness or vomiting to the end of gestation. To the second case the caustic was applied twice only. Improvement followed the first, and complete relief the second application. The third patient required but one application; it was used more freely than in the preceding cases, and applied to the os and a portion of the cervix uteri. The fourth patient needed but one application, and this was one of the most harrassing and persistent cases of vomiting that ever came under my care. The stomach rejected everything taken into it, and the patient grew feeble and became so emaciated that she was scarcely able to leave her bed. The caustic in this case was very freely applied to the os and vaginal cervix. The relief obtained was beyond my expectation, for it was almost immediate. She vomited only twice or thrice in the thirty-six hours following, and no more after that time. She was able to retain food; assimilation was good, and she gained rapidly in health, strength and flesh. The fifth case was one in which the vomiting was not so frequent, but quite as persistent. In this case, in addition to the vomiting, the abdomen was quite tender—as I supposed, from the violent retching. The caustic in this case was applied twice before entire relief was obtained.

In all these cases, before resorting to the caustic, I had faithfully tried, and for some time, remedies which are usually resorted to in such cases, without any benefit whatever in the fourth and fifth cases and only temporary improvement in the others. These were all cases of first pregnancy, except the second one. In the first and second there was slight erosion of the mucous lining around the os; in the others none whatever, all three being perfectly healthy in appearance.

*Notes of a Case, by Dr. Marion Sims.*—I had the good fortune to meet Dr. Jones, of Chicago, last June, when he incidentally related to me his experience in the treatment of the vomiting of pregnancy. I thought the matter of so much importance that I begged him to write it out for publication. Accordingly he sent me the foregoing paper, which I received just as I was leaving for home, and not having time to arrange for its publication there, I now send it to the *Lancet.* I am not in the way of seeing much of this affection, but a case came under my observation a few days ago so strongly confirmatory of Dr. Jones' views, that I take the liberty of appending it to his paper.

Madame de C——, aged twenty-two, married at sixteen, was a very delicate child, but is now a tall, handsome woman, weighing 175 pounds. She has one child, four years and a half old. During her pregnancy she suffered from nausea for two months or more, but not enough to cause anxiety about herself, and she was safely delivered at the full term. She did not nurse the child, and conception occurred again a year after its birth. Nausea began with conception, and continued unabated for two months, when she miscarried. This was at Arcachod, in 1874. In 1875 she conceived again. Conception was immediately followed by nausea, which persisted in spite of the usual remedies, and she miscarried again at the end of the second month. This was at Havre. In 1876 she miscarried a third time in New York, at the end of two months from the prostration of nausea, which began, as before, at the time of conception. She had the ablest counsel in New York—namely, Dr. Wm. Jones, Dr. Thos. F. Cock, and Professor Barker. Her life was in great danger at each of these miscarriages; and the distinguished accoucheur, Professor Fordyce Barker, told her she would hardly survive another such trial as she had just passed through.

I saw Madame de C—— on Oct. 24, 1877. She gave me the history of her miscarriages, and said she feared she was pregnant again. She had just missed her period, and for the last ten days had felt such nausea and disgust for food that she was sure she was pregnant. I gave her some bismuth to take during the day and some bromide of sodium at night. She returned on the 29th, complaining more than ever of nausea, and I prescribed oxalate of cerium. Four days after this Madame de C—— sent for me. She had been confined to her bed for four days, so nauseated that she could not get up, and could not take any nourishment whatever. She did not vomit, but she was completely prostrated by the constant nausea and starvation. She was so changed in appearance since I last saw her that I thought there must be something more the matter with her than the mere nausea of pregnancy. Was it malarial? She had just moved into a new house. Her little boy had been complaining for several days, and her maid-servant had some malarial symptoms requiring quinine. Madame de C—— had lived in malarial regions in America, and she imagined herself worse on alternate days. Thinking there might be a malarial element in her case, I ventured to give her ten grains of quinine in two doses, which unfortunately produced both vomiting and purging, and greatly augmented her prostration. She was now worse than ever. She had had no sleep for two or three nights, and was altogether in a most miserable plight. So I concluded to quiet both stomach and nervous system by bromides, and gave her 120 grains between 5 P.M. and 2 A.M. But she did not sleep, and her condition was now such as to alarm the family. They were evidently as much dissatisfied with my empirical treatment as I was myself. Beginning, at last, to look upon the case as one purely of nausea from pregnancy, I determined to try local treatment.

There was right lateral antiflexion. Both lips of the os tincæ were granular, and covered with a profuse glutinous leucorrhœal secretion. It was a case in which Dr. Graily Hewitt's pessary treatment might have been tried, or Dr. Copeman's plan of forcible dilatation of the os and cervix. The os had been considerably lacerated bilaterally during her labor. The anterior lip was everted as well as eroded, and the finger could easily have been carried into the canal. But having previously made up my mind to try Dr. Jones' method, I cleared away the leucorrhœal discharge, and applied a solution of the nitrate of silver (two drachms to one ounce) freely over the whole surface of the cervix till it was well whitened, and I stopped all other medication. On the next day I found Madame de C—— sitting up in bed, and as bright and cheerful as possible. The change in her voice and general appearance was marvelous. She had had a good night's sleep, the first for a week. She had taken a liberal breakfast, the first good meal for a fortnight, and altogether she felt herself a new being compared with what she was the day before. A show of blood followed the application of the nitrate of silver, and she began to hope that it was a real menstruation. At the end of five or six days there was some nausea, but not at all distressing, and I penciled the neck of the womb with pure carbolic acid till it was completely enveloped in a whitish film. On the next day she said she was perfectly well. On Nov. 19 she came to see me, saying that family affairs called her to New York, and she wished to have the carbolic acid applied again as a precautionary measure. She had occasional nausea, but it amounted to nothing. It did not prevent her from sleeping, and did not prevent her from eating. She had never felt so well before during the first two months of any of her pregnancies.

If Dr. Jones' treatment acts as promptly in all other cases as it did in mine, the profession will certainly feel grateful to him for it.

